# PurUUpurU: An Oligonucleotide Virulence Factor in RNA Viruses

**DOI:** 10.7759/cureus.29340

**Published:** 2022-09-19

**Authors:** Won J Sohn, Gregory D Sloop, Gheorghe Pop, Joseph J Weidman, John A St. Cyr

**Affiliations:** 1 Neurology, University of California, Irvine, Irvine, USA; 2 Pathology, Idaho College of Osteopathic Medicine, Meridian, USA; 3 Cardiology, Radboud University Medical Center, Nijmegen, NLD; 4 Internal Medicine, Independent Research, Columbia, USA; 5 Cardiac/Thoracic/Vascular Surgery, Jacqmar, Inc., Minneapolis, USA

**Keywords:** pathogenesis, cytokines, blood hyperviscosity, inflammation, innate immunity, ebola virus, single-strand rna virus

## Abstract

Background

The copy number of the oligonucleotide 5’-purine-uridine-uridine-purine-uridine-3’ (purUUpurU) motif in a viral genome was previously shown to correlate with the severity of acute illness. This study aimed to determine whether purUUpurU content correlates with virulence in other single-strand RNA (ssRNA) viruses that vary in clinical severity.

Methodology

We determined the copy number of purUUpurU in the genomes of two subtypes of human respiratory syncytial virus (RSV), respiratory syncytial virus A (RSV-A), and respiratory syncytial virus B (RSV-B), which vary in clinical severity. In addition, we determined the purUUpurU content of the four ebolaviruses that cause human disease, dengue virus, rabies virus, human rhinovirus-A, poliovirus type 1, astrovirus, rubella, yellow fever virus, and measles virus. Viral nucleotide sequence files were downloaded from the National Center for Biotechnology Information (NCBI)/National Institutes of Health website. In addition, we determined the cumulative case fatality rate of 20 epidemics of the Ebola virus and compared it with that of the other human ebolaviruses.

Results

The genomic purUUpurU content correlated with the severity of acute illness caused by both subtypes of RSV and human ebolaviruses. The lowest purUUpurU content was in the genome of the rubella virus, which causes mild disease.

Conclusions

The quantity of genomic purUUpurU is a virulence factor in ssRNA viruses. Blood hyperviscosity is one mechanism by which purUUpurU causes pathology. Comparative quantitative genomic analysis for purUUpurU will be helpful in estimating the risk posed by emergent ssRNA viruses.

## Introduction

We recently reported that the number of copies of the oligonucleotide 5’-purine-uridine-uridine-purine-uridine-3’ (purUUpurU) within the genome of several single-strand RNA (ssRNA) viruses correlates with the potential severity of acute illness caused by several of those viruses [1]. ssRNA viruses are a large group [2] that includes important pathogens such as human immunodeficiency virus-1 (HIV-1), hepatitis C virus (HCV), and the coronaviruses that cause severe acute respiratory syndrome (SARS), Middle East respiratory syndrome (MERS), and coronavirus disease 2019 (COVID-19). Coronaviruses have a high copy number of purUUpurU and can cause a fatal acute illness, cytokine storm syndrome, and blood hyperviscosity [1]. In contrast, HIV-1 and HCV have a smaller number of purUUpurU. Those viruses cause only a mild acute illness. In contrast, chronic infection with these viruses is potentially fatal. In general, the severity of acute illness caused by an ssRNA virus correlates with the number of purUUpurU. In our previous study, we identified an exception to this generalization, influenza, which can cause a fatal acute illness even though it has only a moderate copy number of purUUpurU in its genome. We call the process of comparing the number of copies of a nucleotide sequence in different viral genomes quantitative comparative genomic analysis.

PurUUpurU activates innate immunity by binding to Toll-like receptor 8 (TLR8). Acting through the adaptor protein myeloid differentiation primary response 88 (MYD88) and nuclear factor kappa B (NF-κB), this causes the production of the proinflammatory cytokines tumor necrosis factor-alpha (TNF-α), interleukin-1 (IL-1), and interleukin-6 (IL-6) [3]. Both TNF-α and IL-1 cause acute inflammation and upregulate the expression of IL-6. Among other activities, IL-6 activates hepatic production of all components of the acute phase response, including fibrinogen. This increases blood viscosity, turning inflammation into a hyperviscous state and contributing to thromboinflammation [4].

This paper will report the findings of quantitative comparative genomic analysis for purUUpurU in several other viruses. First, we examine two subtypes of respiratory syncytial virus (RSV) that differ in the severity of the illness they cause. In our previous report, we noted a high copy number of purUUpurU in RSV, which can cause a fatal acute illness in infants and the elderly. There are two major subtypes of RSV, respiratory syncytial virus A (RSV-A) and respiratory syncytial virus B (RSV-B). These differ in the severity of illness they cause, with RSV-A causing more severe disease [5-9]. Thus, subtype analysis of RSV provides an opportunity to validate the pathogenicity of purUUpurU.

Next, we will examine the species of ebola virus that cause disease in humans. Epidemics of these viruses, namely, Ebola virus (EBOV), Sudan virus (SUDV), Bundibugyo virus (BDBV), and Taï Forest virus (TAFV), differ in their case fatality rate. Thus, they provide another opportunity to test the pathogenicity of purUUpurU. Finally, we will also report the results of quantitative comparative genomic analysis of other viruses that cause acute illnesses of varying severity, namely, yellow fever virus (YFV), rabies virus, measles virus, human rhinovirus A (HRV-A), astrovirus, rubella, dengue virus, and poliovirus 1.

## Materials and methods

Viral nucleotide sequence files were downloaded from the National Center for Biotechnology Information (NCBI)/National Institutes of Health website (https://www.ncbi.nlm.nih.gov/labs/virus/) in fasta format. All complete (non-partial) nucleotide sequences cumulatively available at the time of analysis were studied. Several curations were performed to remove incomplete sequences. All data analyses were conducted in Python programming language (Python3.7) and Bio.Seq, SeqIO, motifs modules of Biopython toolbox (https://biopython.org/).

The purUUpurU motifs were counted in the following manner: because purine bases are either adenine (A) or guanine (G), the possible purUUpurU motifs are AUUAU, AUUGU, GUUGU, and GUUAU. The complementary DNA sequences are ATTAT, ATTGT, GTTGT, and GTTAT. The strand present in viral particles (i.e., positive or negative) was used for analysis.

If two motifs overlapped, both motifs were tracked. The results report the non-overlapping motif counts with the incidence rate of the overlap. For instance, AUUGUUGU would be detected as two motifs, AUUGU and GUUGU, but there is one non-overlapping motif. The following viruses were examined: RSV-A, RSV-B, BDBV, SUDV, TAFV, EBOV, measles virus, HRV-A, dengue virus, rabies virus, astrovirus, rubella, poliovirus type 1, and YFV.

Definitive outcome data from the West Africa 2013-2016 EBOV epidemic were used to calculate the fatality of that epidemic. Those data were taken from a published source and collected by the World Health Organization (WHO) during the earliest phase of that epidemic, from December 2013 to September 2014 [10]. Fatality data from the other ebolavirus outbreaks (EBOV, n = 19; other ebolaviruses: SUDV, n = 5; BDBV, n = 2; TAFV, n = 1) are calculated using the data obtained by the WHO [11].

The mean number of purUUpurU and genome size (i.e., the number of nucleotides in each genome) were calculated. The coefficient of variation (CV) of both was also calculated. Pearson’s correlation coefficient was used to determine the correlation between genome size and purUUpurU count. This statistic was performed using Microsoft Excel (Redmond, WA, USA). The Mann-Whitney U test was used to determine the significance of the difference between the purUUpurU counts of RSV-A and RSV-B and EBOV and BDVB. A chi-square test was used to determine whether there was a significant difference between the case fatality rates of EBOV and SUDV. These calculations were performed with the Python statistics package (SciPy.stats from SciPy 1.5.2).

## Results

**Figure 1:**
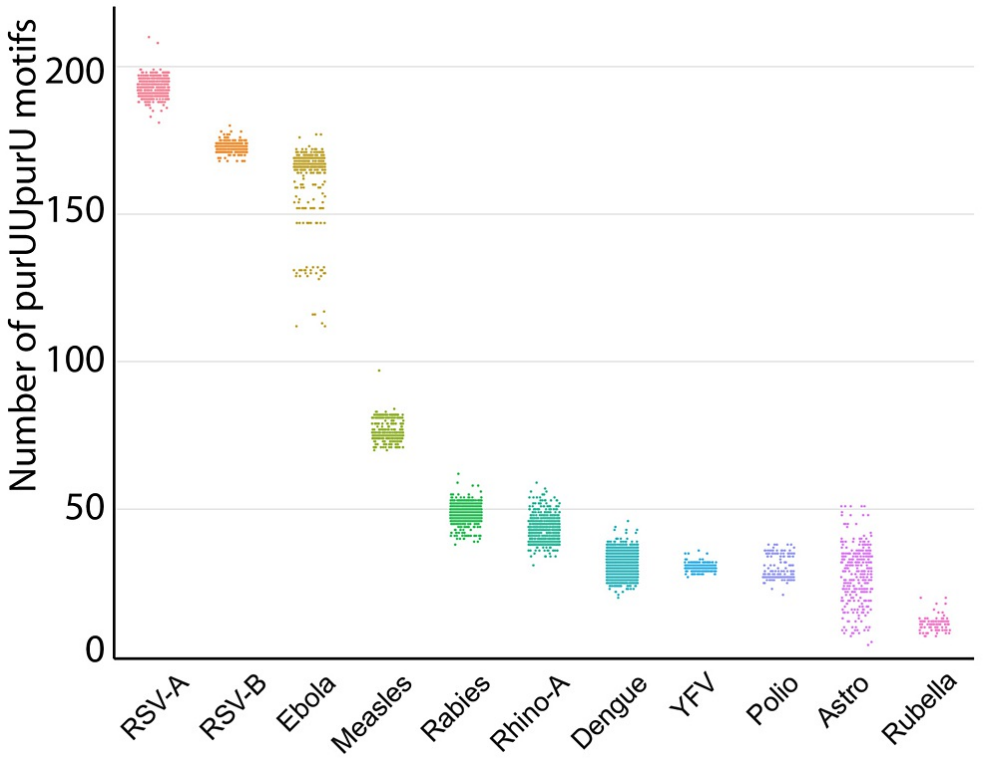
PurUUpurU counts. The four human ebolavirus species (Bundibugyo virus, Sudan virus, Tai Forest virus, and Ebola virus) are grouped as “Ebola” in this figure.  RSV-A, RSV-B, and ebolavirus sp. have a considerably higher purUUpurU content in their genomes than the other viruses. Distinct populations of Ebolavirus, measles virus, rabies virus, and poliovirus 1 can be observed at this level of definition. The strip plot shows the distribution of copy counts of purUUpurU in the all-time data of the viruses. RSV-A = respiratory syncytial virus, type A; RSV-B = respiratory syncytial virus, type B; YFV = yellow fever virus; purUUpurU = 5’-purine-uridine-uridine-purine-uridine-3’

Results of the purUUpurU counting are presented in Figure 1 and Table 1. Ebolavirus species were combined in these. For all viruses, there was a significant correlation between purUUpurU count and genome size (r = 0.80). The greatest number of purUUpurU was in RSV-A, followed by RSV-B, EBOV, measles virus, rabies virus, HRV-A, dengue virus, YFV, poliovirus 1, astrovirus, and rubella virus.

**Table 1:** The results of quantitative comparative genomic analysis for purUUpurU. The four ebolavirus species (Bundibugyo virus, Sudan virus, Tai Forest virus, and Ebola virus) are grouped as “Ebola.” CV = coefficient of variation; RSV-A = respiratory syncytial virus, type A; RSV-B = respiratory syncytial virus, type B; HRV-A = human rhinovirus, type A; YFV = yellow fever virus; purUUpurU = 5’-purine-uridine-uridine-purine-uridine-3’

Virus	Number of purUUpurU			Genome size (number of nucleotides)		Number of genomes analyzed (n)
	Mean	CV (%)	Overlap (%)	Mean	CV (%)	
RSV-A	192.8	1.6	7.7	15,193.0	0.54	526
RSV-B	172.7	0.9	7.6	15,239.8	0.33	377
Ebola	164.8	5.6	4.9	18,908.7	0.72	655
Measles	76.3	4.2	6.1	15,732.5	2.0	433
Rabies	48.2	5.2	1.2	11,914.6	0.59	1592
HRV-A	42.5	9.9	4.1	7,071.7	0.75	756
Dengue	32.0	11	3.7	10,661.6	0.81	4024
YFV	30.4	4.0	2.6	10,850.3	2.16	268
Poliovirus 1	29.2	12	9.0	7,414.9	0.50	225
Astrovirus	28.1	33	4.0	6,657.6	10.9	340
Rubella	11.3	22	0.1	9,761.6	0.07	89

**Figure 2:**
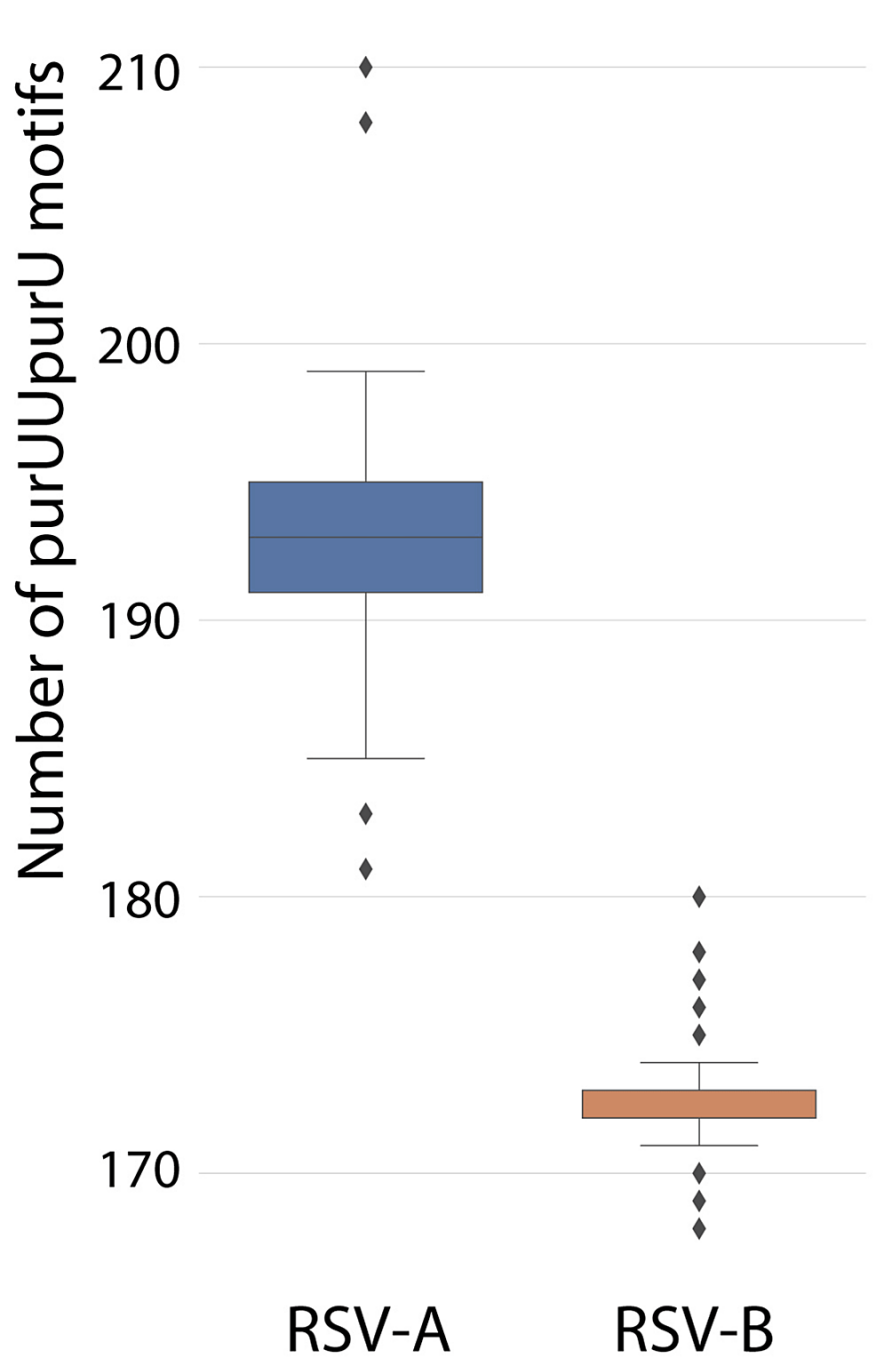
A significant difference in the number of purUUpurU between RSV-A and RSV-B. The boxes define the interquartile range (IQR) between the 25th and 75th percentiles. The boundary whiskers are located 1.5 IQR values away from the box. The diamond shapes represent outliers beyond the boundaries of the whiskers. RSV-A = respiratory syncytial virus, type A; RSV-B = respiratory syncytial virus, type B; purUUpurU = 5’-purine-uridine-uridine-purine-uridine-3’

The purUUpurU count was a strong discriminant between the RSV-A and RSV-B (Figure 2). The genome of RSV-A contains more purUUpurU than the genome of RSV-B, 192.8 versus 172.7 (Mann-Whitney U test, U = 0.0, p < 0.000001).

**Figure 3:**
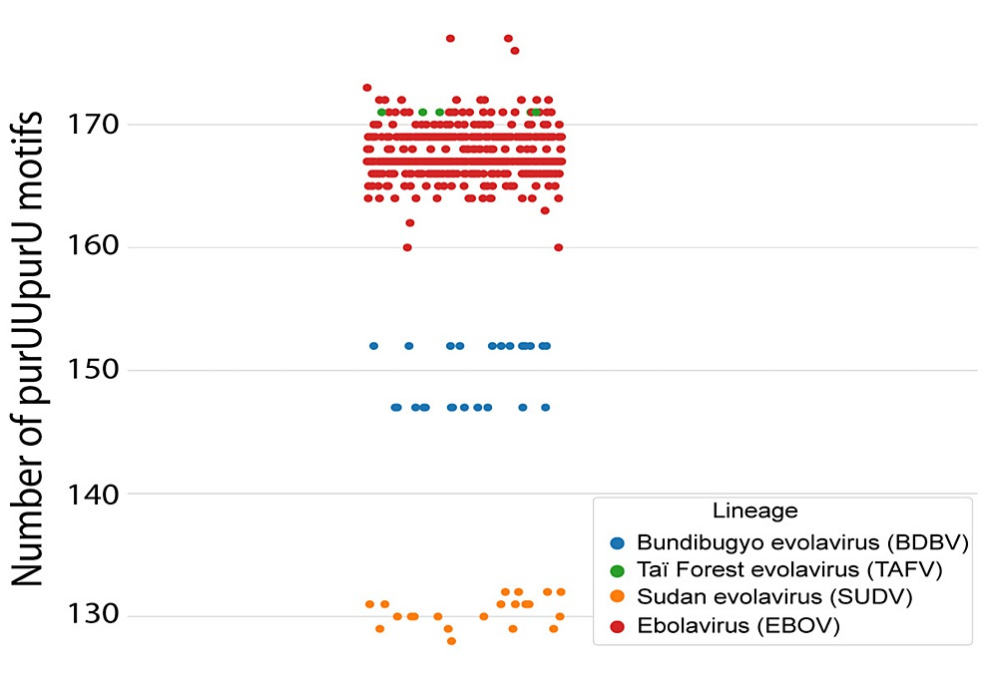
Representation of the purUUpurU counts in the species of ebolavirus that cause disease in humans: Bundibugyo virus, Sudan virus, Tai Forest virus, and Ebola virus. The swarm plot shows no overlap in purUUpurU content except between the Ebola virus and the Tai Forest virus. purUUpurU = 5’-purine-uridine-uridine-purine-uridine-3’

Figure 1 shows that the purUUpurU content defines distinct subtypes of ebola virus, measles virus, rabies virus, and polio virus 1. These are visible as distinct bands in a more uniform population. PurUUpurU counts were more uniformly distributed in the dengue virus and astrovirus genomes. The bands in ebola virus prompted further examination. Data regarding the individual ebola viruses are presented in Table 2. The genome of EBOV contained significantly more purUUpurU than BDBV, 167.6 versus 149.8 (Mann-Whitney U test, U = 0.0, p < 0.000001). Figure 3 shows the distribution of purUUpurU in the four ebola virus species which cause human disease, namely, BDBV, SUDV, TAFV, and EBOV. 

**Table 2:** The results of quantitative comparative genomic analysis for purUUpur in the ebolaviruses that cause disease in humans. CV = coefficient of variation; purUUpurU = 5’-purine-uridine-uridine-purine-uridine-3’

Virus	Number of purUUpurU		Genome size (number of nucleotides		Number of genomes analyzed (n)
	Mean	CV (%)	Mean	CV (%)	
Ebola virus (EBOV)	167.6	1.07	18,922.7	0.61	574
Sudan virus (SUDV)	130.4	0.86	18,864.0	0.09	23
Bundibugyo virus (BDBV)	149.5	1.71	18,635.0	1.66	26
Taï Forest virus (TAFV)	171.0	0	18,923.8	0.12	4

The mean purUUpurU count decreased in the following order: TAFV > EBOV > BDBV > SUDV. Case fatality rates were as follows: EBOV, 69.2% (6,892 cases, 4,772 deaths in cases with definitive outcomes), SUDV, 53.7% (784 cases, 421 deaths), BDBV, 32.0% (103 cases, 33 deaths), and TAFV, 0 deaths (one case). Using these data, the case fatality rate for the disease caused by EBOV was higher than for the disease caused by SUDV (chi-square = 77.7, p < 1e-10).

The percentages of overlapping motifs ranged from 0.1% (rubella virus) up to 9.0% (poliovirus 1) (Table 1). The potential significance of the rate of overlap was not assessed in this study.

## Discussion

Respiratory syncytial virus and increased blood viscosity

The mean number of purUUpurU motifs in the genomes of RSV-A and RSV-B correlates with the severity of the disease they cause. The greater number of purUUpurU could cause more severe disease through multiple mechanisms. The greater number of purUUpurU in RSV-A could result in higher elevations of TNF-ɑ, which has been associated with more severe bronchospasm [12]. The greater number of purUUpurU could also stimulate greater elevations of IL-6. Plasma levels of IL-6 correlate with disease severity in RSV [13].

IL-6 upregulates hepatic expression of acute phase reactants and decreases the synthesis of albumin. By increasing plasma concentrations of large proteins such as fibrinogen and decreasing the albumin concentrations, the acute phase reaction increases blood viscosity [5]. Driven by the high copy number of purUUpurU in the genome of severe acute respiratory syndrome coronavirus 2 (SARS-CoV-2) [1], blood viscosity in COVID-19 was increased by 17.6% in a study of 15 patients hospitalized in an intensive care unit [14]. An increase in blood viscosity causes a threefold inverse change in tissue perfusion [15]. By decreasing perfusion, elevated blood viscosity caused by viral-induced acute inflammation can cause organ dysfunction independent of direct viral invasion.

Elevated blood viscosity may explain many of the clinical features that are more severe in RSV-A, such as degree of oxygen desaturation, acidosis, hypercapnia, the prevalence of apnea in the hospital, length of hospitalization, and the need for mechanical ventilation [9]. Decreased pulmonary blood flow will reduce gas exchange, causing hypoxia and hypercapnia. Thus, increased blood viscosity and subsequent decreased blood flow can cause both metabolic and respiratory acidosis. Decreased perfusion of the respiratory center in the brainstem will reduce its activity. Hypoxia will further impair the function of this and all other tissues and organs. This can increase the chance of apnea, particularly in the immature central nervous system. These will increase the need for mechanical ventilation and prolong hospitalization.

Ebolaviruses

These data also suggest that genomic purUUpurU content is a virulence factor in diseases caused by EBOV and other ebolaviruses. Consistent with a larger number of purUUpurU, epidemics of EBOV are associated with a higher case fatality rate than epidemics caused by the other ebolaviruses. As with all other viral diseases discussed in this paper, ebolavirus disease begins with nonspecific symptoms due to circulating cytokines, i.e., high fever, malaise, loss of appetite, etc. The synthesis of these cytokines is driven, at least in part, by purUUpurU. Severe ebolavirus disease can progress to multiorgan failure, particularly involving the gastrointestinal tract, liver, brain, and kidneys. Similar to cytokine storm in SARS-CoV-2, this has been attributed to “immune dysregulation” [16]. However, it is possible that increased levels of cytokines driven by the high content of purUUpurU in EBOV, and to a lesser extent the other ebolaviruses, contribute to severe disease. Viral invasion undoubtedly plays a role in the more specific symptoms caused by EBOV and other ebolaviruses.

Excluding the West Africa outbreak of 2013-2016, the case fatality rate from the 19 other EBOV outbreaks ranged from 42% to 90% with an average of 68.7% (5,155 cases, 3,542 deaths) [11]. Based on definitive outcomes, the case fatality rate during the first nine months of the West Africa outbreak was 70.8% [10]. However, based on incomplete data, the case fatality rate was estimated to be only 37.7%. This figure is inaccurate for many reasons; for example, it included patients who were alive at the time of data collection and later died. The authors noted that their data were collected in extreme conditions and that the priorities during an epidemic are patient care and controlling the epidemic, not epidemiologic investigations. Thus, the authors considered their projections imprecise. At this time, the WHO website shows a case fatality rate of 39.5% for the 2013-2016 epidemic [11]. Those are the only data that are marked with an asterisk to indicate the totals including suspected, probable, and definitive cases.

Nevertheless, very similar figures are cited in the literature [16-18]. This outbreak comprises the majority of the total estimated EBOV cases as of 2021, 33,501 [17]. Based on this estimation, the authors of a recent review wrote: “On the basis of comparative statistics on CFRs (case-fatality rates), a fundamental difference in virulence between ebolaviruses that cause lethal human disease is not observed; the oft-repeated notion that EBOV is the most virulent ebolavirus (let alone filovirus) is not supported by available data. The mean CFRs for each ebolavirus are 33.65 ± 8.38% (BDBV), 43.92 ± 0.7% (EBOV) and 53.72 ± 4.456% (SUDV); that is, a CFR of ~40-50% overall, with the remaining difference between the viruses compounded by the number of outbreaks recorded and the typically small number of cases in each outbreak. Accordingly, whether one ebolavirus is more dangerous than another is statistically unclear” [16].

**Figure 4:**
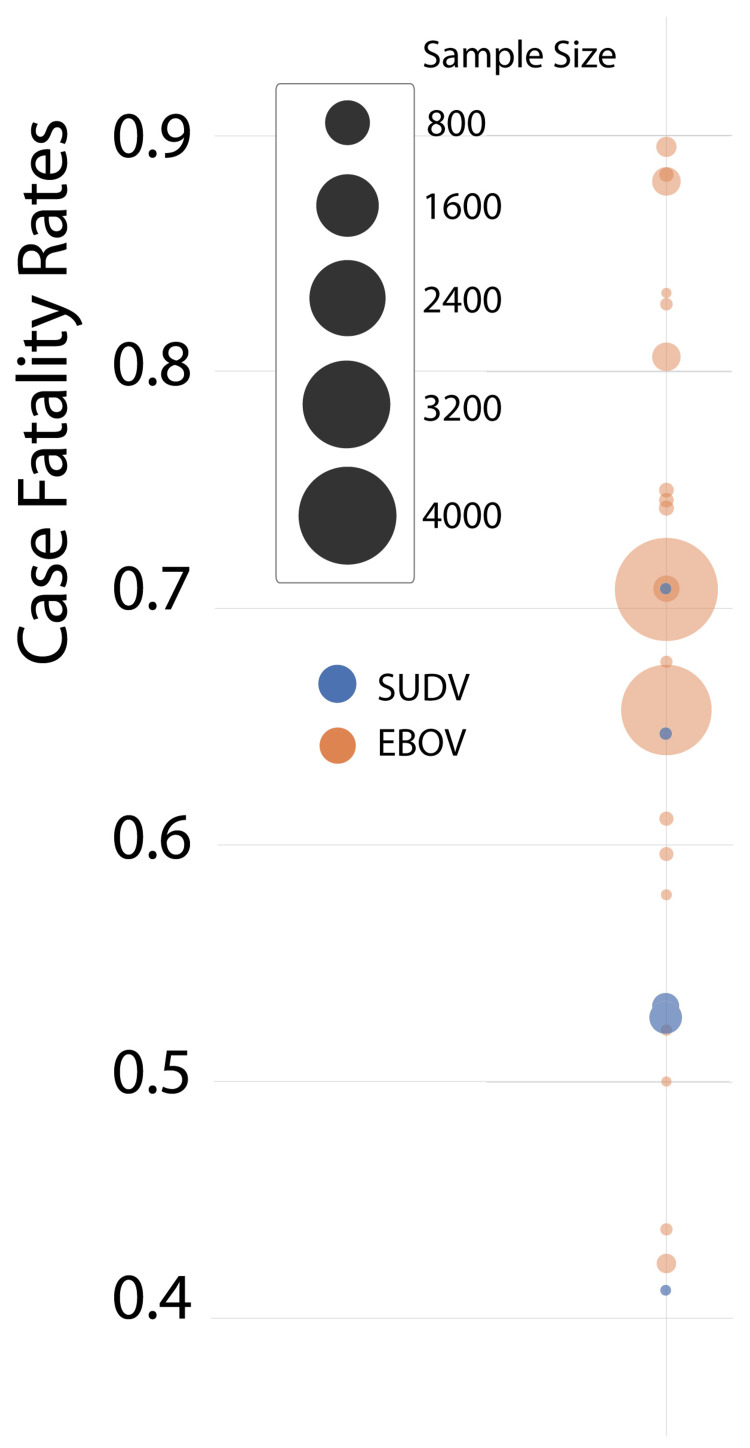
Representation of the case fatality rate of the epidemics caused by the EBOV and the SUDV. The size of the circles reflects the size of the population in the respective Ebola studies. Data from [11] used under CC BY-NC-SA 3.0 IGO (Creative Commons Attribution-NonCommercial-ShareAlike 3.0 IGO). EBOV = Ebola virus; SUDV = Sudan virus

Elucidation of genomic purUUpurU content as a virulence factor provides a biological basis for a higher case fatality rate in epidemics caused by EBOV. Further, a case fatality rate of 39.5% is lower than any other epidemic caused by EBOV. Thus, we regard data from the definitive outcomes of smaller outbreaks and the beginning of the West Africa 2013-2016 outbreak to be the most reliable and that EBOV is the most virulent ebolavirus (Figure 4).

Dengue virus

The moderate copy number of purUUpurU in the genome of the dengue virus may seem surprising given its association with severe diseases such as “break-bone fever” and viral hemorrhagic disease. However, of the estimated 390 million annual dengue virus infections, 293.9 million are estimated to be clinically inapparent [19]. Severe illness caused by the dengue virus is usually seen in secondary heterotypic infections, i.e., when the patient is infected by a different serotype from the initial infection. In this situation, non-neutralizing antibodies allow viruses to be phagocytized and infect cells that bear receptors to the fragment crystallizable (Fc) portion of antibody molecules in the process called antibody-dependent enhancement [2]. Thus, acquired immunity, not innate immunity, is the principal cause of severe disease produced by the dengue virus.

Nonspecific viral syndromes

At both ends of the spectrum, in many cases, the genomic purUUpurU content accounts for the potential severity of acute illness caused by the virus. SARS-CoV-2 has 247 copies of purUUpurU in its genome [1] and can cause a fatal acute illness characterized by cytokine storm syndrome and complications caused by extreme blood hyperviscosity. At the other end of the spectrum, rubella, with 11 purUUpurU, causes a relatively harmless, mildly febrile illness [2]. In viruses with moderate content of purUUpurU, the oligonucleotide drives the initial synthesis of cytokines that cause a nonspecific viral syndrome. Specific symptoms caused by these viruses depend on viral tropism, e.g., encephalitis in rabies, rhinitis in HRV-A, etc. These symptoms, as well as severe disease in dengue and influenza A, are clearly not driven by purUUpurU.

Significance of PurUUpurU

PurUUpurU can be viewed as a viral equivalent of N-formylmethionine, a product of bacterial metabolism that activates innate immunity. It is a driver of the cytokines that cause the symptoms seen in the initial nonspecific phase of many viral illnesses. If the infection progresses, continued production of cytokines driven by purUUpurU can lead to elevated blood viscosity with subsequent organ dysfunction due to reduced tissue perfusion, as seen in the disease caused by RSV and ebolaviruses. This outcome is likelier if the host interferon response is deficient. In infections caused by viruses with the greatest purUUpurU content, inflammation driven by that oligonucleotide can lead to marked elevations of cytokines, hyperviscosity syndrome, and thromboinflammation. The high copy number of purUUpurU in SARS-CoV-2 suggests that the cytokine storm seen in COVID-19 is caused by the virus, not dysregulation of the host immune system. This may also be the case in the disease caused by EBOV. Thus, the purUUpurU content of a viral genome influences the inflammatory potential of that virus.

Quantitative comparative genomic analysis has contributed to the understanding of how much of the variation in a clinical outcome is determined by the virus and how much is due to variation in the host immune response. PurUUpurU data suggest that viruses can cause cytokine storm syndrome in a normally functioning immune system. Quantitative comparative genomic analysis has revealed that the genomic purUUpurU content is an important characteristic of any ssRNA virus. A 2007 publication reported that 49 of the 87 new human pathogens identified since 1980 are RNA viruses [20]. Knowing the purUUpurU content of an emerging RNA virus will be important in assessing the threat posed by that virus.

**Figure 5:**
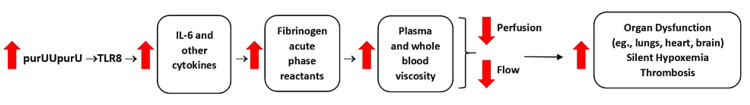
The mechanism by which purUUpurU causes hyperviscosity, decreased tissue perfusion, organ dysfunction, and thrombosis. TLR8 = toll-like receptor 8; IL-6 = interleukin 6; purUUpurU = 5’-purine-uridine-uridine-purine-uridine-3’ Reprinted from reference [1] under Creative Commons BY 4.0.

Finally, this work highlights the pathogenic role of blood hyperviscosity in diseases. Elucidation of one driver of cytokine elevations in severe RNA viral infections allows informed speculation of how those cytokines cause disease. By upregulating the synthesis of acute phase reactants such as fibrinogen, a high copy number of purUUpurU can lead to extraordinary elevations of blood viscosity, thereby decreasing tissue perfusion and increasing the risk of thrombosis (Figure 5) [1]. Prior to the elucidation of the role of purUUpurU and hyperviscosity in viral inflammation, the cause of certain complications of COVID-19, such as silent hypoxemia and myocarditis, was poorly understood. These complications can reasonably be attributed to blood hyperviscosity.

A meta-analysis of inflammatory markers in COVID-19 showed that in critically ill patients, acute phase reactants and related biomarkers are elevated to a greater degree than cytokines. The authors questioned the relevance of cytokine storm in organ dysfunction in COVID-19. Instead, the degree of elevation of D-dimer, the degradation product of cross-linked fibrin, was the strongest discriminant between critically ill COVID-19 patients and those with sepsis [21]. Increased blood viscosity predisposes to thrombosis and, following fibrinolysis, elevations of D-dimers (Figure 5).

Along with impaired myocardial contractility and capillary leak, blood hyperviscosity should be recognized as a severe adverse effect of marked cytokine elevations. All of these factors decrease tissue perfusion. Cytokine elevations change from being beneficial to pathologic when they cause decreased tissue perfusion in the host.

## Conclusions

The quantity of genomic purUUpurU is a virulence factor in ssRNA viruses. Blood hyperviscosity is one mechanism by which purUUpurU causes pathology. Comparative quantitative genomic analysis for purUUpurU will be helpful in estimating the risk posed by emergent ssRNA viruses.
